# Editorial: Emerging mechanisms in cardiovascular disease

**DOI:** 10.3389/fphar.2023.1301124

**Published:** 2023-10-20

**Authors:** Huseyin C. Yalcin, Elisabetta Caiazzo, Armando Ialenti, Ali H. Eid

**Affiliations:** ^1^ Biomedical Research Center, Qatar University, Doha, Qatar; ^2^ Department of Pharmacy, School of Medicine and Surgery, University of Naples Federico II, Naples, Italy; ^3^ School of Infection and Immunity, College of Medical, Veterinary and Life Sciences, University of Glasgow, Glasgow, United Kingdom; ^4^ Department of Basic Medical Sciences, College of Medicine, QU Health, Qatar University, Doha, Qatar

**Keywords:** cardiovascular disease, phytochemicals, mitochondrial dysfunction, resistance arteries, pre-eclampsia

The leading cause of worldwide mortality is cardiovascular disease (CVD) (Aboukhater et al., 2023). Despite many significant advances in the field, CVD continues to claim more lives than all cancers combined (Sawma et al., 2022). There is then an urgent need for more efficacious treatment modalities or therapeutics that could aid in the management of CVD (Badran et al., 2019; Maaliki et al., 2019; El-Hachem et al., 2021). For such potential new drugs to be determined, a better understanding of the underlying mechanisms and the potential targets is critical. This Research Topic seeks to highlight a few of these emerging mechanisms and targets that could be employed for a better treatment of CVD.

Myocardial injury continues to be a major contributor to CVD-associated mortality. In this thematic issue, Liu et al. discuss how they established a model for coronary microembolization (CME) in rats, and report that ferroptosis and inflammation are two key players in CME-induced myocardial injury. The authors then show that suppressing ferroptosis attenuates myocardial injury and inflammation following CME. It appears that Ptgs2, a core factor in ferroptosis, and Hif1a are the two mediators of this suppressed ferroptosis. Importantly, the authors further report that by inhibiting the Hif1a/Ptgs2 axis, atorvastatin was able to suppress ferroptosis-dependent CME-precipitated myocardial injury and inflammation (Liu et al.).

In a longitudinal study, Cassano et al. report that in patients suffering from heart failure with reduced ejection fraction (HFrEF), sacubitril/valsartan suppressed oxidative stress, inhibited platelet activation, and improved endothelial dysfunction. These findings further support the utilization of sacubitril/valsartan in HFrEF, especially that the beneficial effects reported were noticed after 6 months of having patients on sacubitril/valsartan.

The role of mitochondria in various aspects of the cardiovascular system is becoming clearer and more appreciated. In this Research Topic, Fang et al. show that mitochondrial fission is an emerging player in vascular smooth muscle cell lipid deposition and foaming. The authors also report that stimulation with oxidized low-density lipoprotein (ox-LDL) could drive over-fission, and eventually precipitating dysfunction, of mitochondria (Fang et al.). Interestingly, some natural products are being considered as agents that could reduce the burden of mitochondrial dysfunction. In this Research Topic, Liao et al. discuss how natural compounds could target mitochondrial dysfunction, and how such an approach could be an emerging tactic to treat or ameliorate organ damage in hypertension (Liao et al.). Among these natural compounds are flavonoids and alkaloids, which are known to exert various cardio-vasculoprotective roles (Fardoun et al., 2019; Maaliki et al., 2019; Fardoun et al., 2020; Slika et al., 2022). Likewise, Qishen Yiqi Dripping Pill (QSYQ), a standardized Chinese herbal preparation approved for treating cardiovascular disease, can reduce myocardial injury. Here, Li et al. show how QSYQ ameliorates myocardial ischemia/reperfusion injury, and improves cardiac structure and function by abrogating autophagy and NLRP3 inflammasome.

Nourian et al. report that there is a continuous molecular and structural remodeling of small arteries (mesenteric or cerebral) during the postnatal period of the rat. Importantly, this remodeling is also reflected in the functional responsiveness of these very arteries where the endothelium-dependent hyperpolarization component of dilation is shown to develop postnatally (Nourian et al.) and that dilation occurs in a mechanism that appears to mirror the structural maturation of the internal elastic lamina (Nourian et al.).

Morgaan et al. investigated the impact of pre-eclampsia on the renal and hemodynamic disturbances in endotoxic adult offspring, and whether these perturbations can be ameliorated with the antenatal administration of pioglitazone with or without losartan. Interestingly, this study highlights a role for the sex of the animal in pre-eclamptic fetal programming of hemodynamic and renovascular effects of endotoxemia in the adult offspring of the rat (Morgaan et al.). Moreover, prenatal pioglitazone/losartan therapy appears to be superior to individual therapy in suppressing the inflammatory response in pre-eclamptic rats (Morgaan et al.).

Papers presented in the Research Topic cover a variety of emerging mechanisms for CVD progression. Further research on such mechanisms will pave road for better therapies. Maybe more importantly, several of the papers involved pre-clinical pharmaceutical approaches to suppress investigated cardiac injuries. Knowledge from these works will be critically important for following clinical studies.
